# Pro-neurotrophins secreted from retinal ganglion cell axons are necessary for ephrinA-p75^NTR^-mediated axon guidance

**DOI:** 10.1186/1749-8104-5-30

**Published:** 2010-11-02

**Authors:** Katharine JM Marler, Subathra Poopalasundaram, Emma R Broom, Corinna Wentzel, Uwe Drescher

**Affiliations:** 1 MRC Centre for Developmental Neurobiology, King's College London, New Hunt's House, Guy's Campus, London SE1 1UL, UK; 2 Centre for Neuroendocrinology, UCL, Royal Free Campus, Rowland Hill Street, London NW3 2PF, UK; 3 Friedrich Miescher Institute for Biomedical Research, Maulbeerstrasse 66, PO Box 2543, 4058 Basel, Switzerland

## Abstract

**Background:**

Retinotectal map formation develops via topographically specific guidance and branching of retinal axons in their target area. This process is controlled, in part, by reverse signalling of ephrinAs expressed on retinal axons. As glycosylphosphatidylinositol-anchored molecules, ephrinAs require transmembrane co-receptors to exert this function, for which the two neurotrophin receptors, p75^NTR ^and TrkB, were recently proposed.

**Results:**

We show here that the ligands for these receptors, the brain-derived neurotrophic factor precursor (proBDNF) and its processed form, BDNF, respectively, control the branching of retinal axons antagonistically, which they mediate by inducing the corresponding neurotrophin receptor-ephrinA complexes. Moreover, scavenging proneurotrophins, by adding antibodies specific for the pro-domain of proBNDF or a soluble extracellular domain of p75^NTR^, abolish repellent ephrinA reverse signalling in the stripe assay.

**Conclusions:**

This indicates that retinal cells secrete proneurotrophins, inducing the ephrinA-p75^NTR ^interaction and enabling repellent axon guidance. The antagonistic functions of proBDNF and BDNF raise the possibility that topographic branching is controlled by local control of processing of proneurotrophins.

## Background

The retino-tectal projection is a well suited model system to investigate the formation of topographic maps and the control of local axon branching. In this projection, retinal ganglion cell (RGC) axons grow into the tectum in a non-topographic manner and initially overshoot their future termination zones. Termination zones are formed through interstitial branching, with branching of axons from nasal retina in the caudal tectum and axons from temporal retina in rostral tectum. The map is refined by arborisations and pruning of overshoot axon segments. The final map is a product of both activity-independent and activity-dependent processes [1-3].

Some aspects of this mapping process are controlled by retinally expressed ephrinA molecules, with higher expression on nasal than on temporal retinal axons. This differential expression mediates a repulsion of nasal axons from parts of the target area expressing high(er) amounts of EphA molecules, that is, the anterior tectum [4]. Recently, the neurotrophin receptors p75^NTR ^and tropomyosin-related kinase (Trk)B were proposed as co-receptors for ephrinAs, which are glycosylphosphatidylinositol-anchored and therefore have no direct contact with the cytosol [5,6].

Ligands for these receptors are the brain-derived neurotrophic factor precursor (proBDNF) and its processed form, BDNF. proBDNF binds with high affinity to p75^NTR^, while BDNF binds with high affinity to TrkB [7-9]. Both the pro-form and the processed form are secreted from neurons, and their processing is controlled on various levels [10-17]. This control of processing is crucial, as the activation of either p75^NTR ^or TrkB leads often to opposing biological effects [18]; for example, activation of TrkB results in cell survival, while activation of p75^NTR ^leads to cell death [19]. Similarly, in synapse function, TrkB and p75^NTR ^are involved antagonistically in long-term plasticity versus long-term depression [20,21].

This study investigates whether the interaction of ephrinAs with either TrkB or p75^NTR ^also results in antagonistic effects on axon guidance and branching of retinal axons [22]. We have approached this by using various *in vitro *assays. Our findings on the antagonistic functions of proBDNF versus BDNF on axon guidance and branching fit well to data showing that a (conditional) inactivation of p75^NTR ^results in a disturbance of the retinocollicular map with shifted and ectopic termination zones and an increase in non-topographic branching anterior to the termination zones [5].

## Results and discussion

### Ligand-promoted interaction of ephrinA5 with p75^NTR^

**Figure 1:**
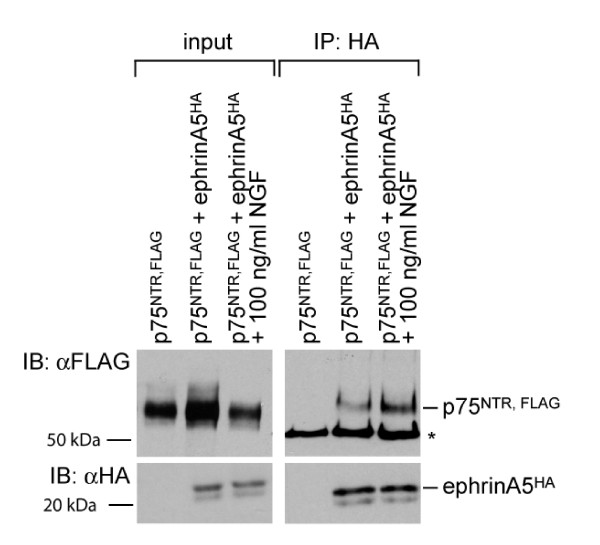
**The ephrinA5-p75^NTR ^interaction is promoted in a ligand-dependent manner**. CHO cells were transfected with cDNA expression vectors for p75^NTR, FLAG ^and ephrinA5^HA. ^A day later cells were serum starved and treated for 30 minutes with 100 ng/ml NGF as indicated. Subsequently, cells were lysed and immunoprecipitated using a αHA antibody. Western blot analyses of input and immunoprecipitate showed that co-immunoprecipitation of p75^NTR ^and ephrinA5 is increased in the presence of ligand. A quantification of these experiments and comparable experiments using proneurotrophins are shown in Additional files 1 and 2. The asterisk marks the Ig heavy chain of the antibody used for immunoprecipitation. IB, immunoblot; IP, immunoprecipitation.

We have recently shown that the interaction between ephrinAs and TrkB is promoted by the ligand BDNF [6]. EphrinAs interact also with p75^NTR ^*in vivo*, though a ligand-dependency was not investigated here [5]. We have here addressed this question by co-transfection of ephrinA5 and p75^NTR ^cDNAs into Chinese hamster ovary (CHO) cells and co-immunoprecipitations before and after activation of p75^NTR ^(Figure 1). Our results show that in the absence of p75^NTR ^activation, only little p75^NTR ^co-immunoprecipitates with ephrinA5, while after its activation the amount of p75^NTR ^was substantially increased (Figure 1). Data from four independent experiments showed an increase in co-immunoprecipitated p75^NTR^. A comparable induction of the p75^NTR^-ephrinA5 complex was observed after application of proBDNF or the precursor of nerve growth factor (proNGF; Additional files 1 and 2). Thus, both the ephrinA-p75^NTR ^and the ephrinA-TrkB interaction are promoted by their respective ligands.

### Proneurotrophin secretion is necessary for repellent ephrinA reverse signalling

**Figure 2:**
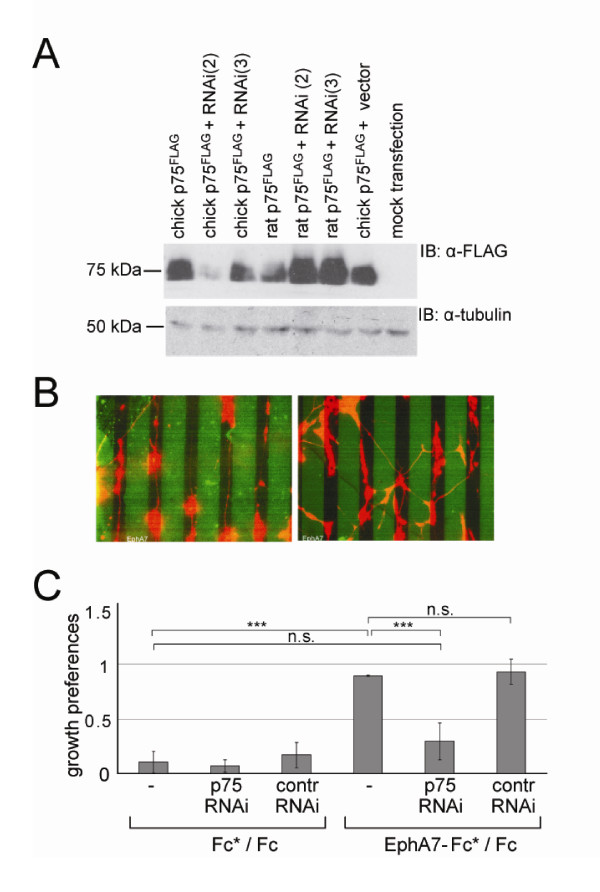
**Guidance of retinal axons on an EphA7-Fc versus Fc substrate is abolished by knockdown of p75^NTR^**. **(A) **Efficiency of selected RNAi experiments to knock down p75^NTR ^protein. Chick or rat p75^NTR, FLAG ^was expressed in CHO cells in parallel to different RNAi experiments targeting selected chick p75^NTR ^sequences. RNAi(2) almost completely knocks down chick but not rat p75^NTR^, while RNAi(3) has no effect on either. α-Tubulin levels are shown for loading controls. **(B) **Single cells from embryonic day 6 nasal thirds of chick retina were electroporated with RNAi vectors for p75^NTR^. These RNAi vectors also contained a red fluorescent protein (RFP) cassette. Then cells were plated on a substrate of alternating lanes of EphA7-Fc and Fc stripes. The first generated stripe was labelled by adding FITC-Fc at low concentrations (indicated by EphA7-Fc*, or Fc* in Fc/Fc controls). In the pictures shown, the green stripes represent the EphA7-Fc stripe. Stripe width is 50 μm. Two days later, outgrowth preferences of retinal axons were analysed. The picture on the left shows the growth preference on EphA7-Fc*/Fc stripes after electroporation of control RNAi(3), and the picture on the right that after electroporation of RNAi(2). Knockdown of p75^NTR ^leads to a strong abolishment of the striped outgrowth of retinal axons. For all stripe assays (and outgrowth/branching assays) the evaluation was done blind to the composition of stripes, constructs transfected and ligands added. **(C) **Quantification of growth preferences after p75^NTR ^knockdown. The results of three independent experiments are shown. Error bars denote standard error of the mean and significance is indicated as ***P < 0.001; n.s., not significant. Statistical analysis was done in GraphPad using one way ANOVA and Tukey *post hoc *test (GraphPad: LaJolla, CA, USA).

In stripe assay experiments using a substrate of alternating lanes of EphA7-Fc and Fc protein overlaid with laminin/merosin, nasal retinal axons are repelled from growing on EphA7-Fc lanes, leading to a striped outgrowth [4]. This matches, in principle, their targeting behaviour *in vivo *(Additional file 3), where axons with high expression of ephrinAs avoid regions in the tectum with high EphA concentrations. In further support of the idea of repellent ephrinA reverse signalling involving p75^NTR^, we show now that RNA interference (RNAi)-mediated knockdown of p75^NTR ^abolishes the striped outgrowth of retinal axons (Figure 2) [5].

Interestingly, these experiments (as well as comparable experiments with retinae from wild-type and p75^NTR ^knockout mice [5]) were performed in the absence of added pro-neurotrophins, which is surprising in view of our data showing a ligand-induced/promoted interaction of ephrinA5 with p75^NTR ^(see above; Figure 1).

**Figure 3:**
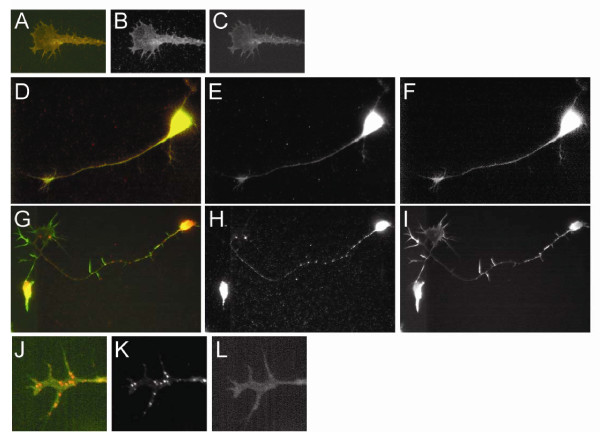
**Expression of proBDNF in chick RGC axons**. Retinal single cell cultures derived from E6 chick retina were stained after 2 days *in vitro *using a monoclonal antibody against the pro-domain of proBDNF [14] according to protocols given in Yang *et al*. [14]. **(A-C) **An RGC growth cone stained with control antibody (B), and phalloidin (C) to show the location of actin. (A) The composite of (B) and (C). In all composites phalloidin is shown in green and proBDNF/control antibody in red. **(D-F) **An RGC axon stained with control antibody (E), and phalloidin (F). (D) The composite of (E) and (F). **(G-I) **An RGC axon stained with a proBDNF antibody (H) [14], and phalloidin (I). (G) The composite of (H) and (I). **(J-L)**. An RGC growth cone stained with proBDNF antibody (K), and phalloidin (L). (J) The composite of (K) and (L).

We hypothesized that proneurotrophins might be secreted from retinal axons and, in an autocrine-paracrine loop, induce the formation of the ephrinA5-p75^NTR ^receptor complex. It had been shown already that chicken RGCs express RNA coding for proBDNF/BDNF starting at embryonic day (E)6 [23] and that BDNF protein is present in the RGC layer at E8.5 [24]. However, the expression of proBDNF was not investigated. Here we have stained explant cultures from chick E6 retinae with a well characterised antibody raised against the pro-domain of proBDNF [14] to show the presence of this protein in axons and growth cones of RGCs (Figure 3).

**Figure 4:**
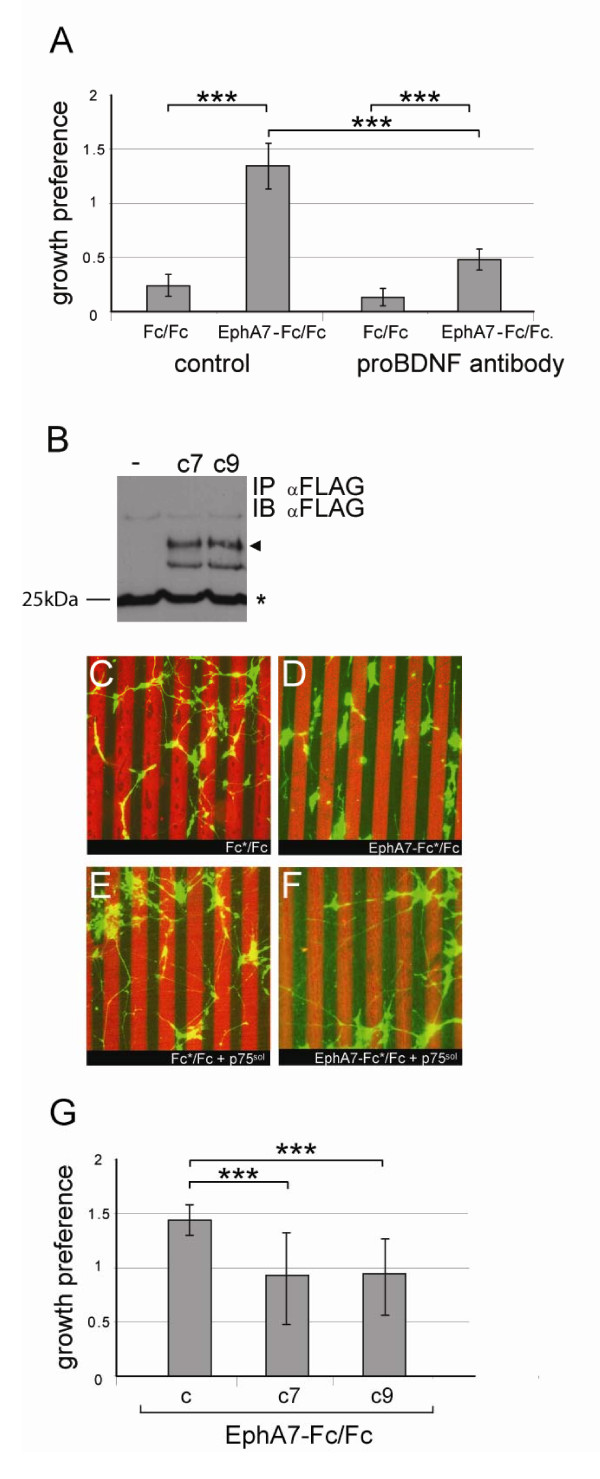
**Repellent axon guidance is disrupted in the presence of an anti-proBDNF antibody or a soluble extracellular domain of p75^NTR^**. **(A) **Quantification of axon growth preferences in the presence of an anti-proBDNF antibody [14] or a control antibody. Stripe assay experiments were performed in the presence of a proBDNF antibody (1:200) [14] or a control antibody (mouse monoclonal antibody for placental alkaline phosphatase; 1:200). The quantification of axon growth preferences shows an abolishment of repellent guidance in the presence of the proBDNF antibody (see also Additional file 4). Error bars represent the standard error of the mean. Statistics were performed using Kruskal-Wallis test and Dunn's multiple comparison test with ***P < 0.001. **(B) **Immunoprecipitation and western blot analysis of CHO cell supernatants transfected with p75^NTR, sol, FLAG ^analysing two different clones (c7 and c9). The arrowhead points to the band corresponding to p75^NTR, sol, FLAG^; the asterisk indicates the Ig light chain from the αFLAG antibody. The first lane shows the analysis of mock-transfected cells. IB, immunoblot; IP, immunoprecipitation. **(C-F) **Single cells from E6 retina were electroporated with an eGFP expression plasmid and plated on alternating lanes of EphA7-Fc/Fc, or Fc/Fc (see (A)). Two days later, the cultures were fixed and analysed for their growth preferences. (C, E) Fc versus Fc stripes. (D, F) EphA7-Fc versus Fc stripes. (C, D) Addition of media from mock transfected CHO cells. (E, F) Addition of media from CHO cells transfected with an expression plasmid for the extracellular soluble domain of p75^NTR ^(p75^NTR, sol^). **(G) **Quantification of axon growth preferences in the presence of p75^NTR, sol ^or control medium. Two different p75^NTR, sol, FLAG ^clones (c7 and c9) were analysed and led to a similar reduction in the growth preference of RGC axons. Statistics were performed in GraphPad using Kruskal-Wallis with *post hoc *Dunn's multiple comparison test.

To test functionally the secretion of proBDNF from retinal cells (inducing an ephrinA-p75^NTR ^complex), we added the antibody against the pro-domain of proBDNF (see above and [14]) to the medium of the stripe assay experiments as a scavenger to neutralise any secreted proBDNF. We found that the presence of this antibody led to the loss of striped outgrowth, indicating abolishment of repulsion of retinal axons from EphA7-Fc lanes (Figure 4A; Additional file 4). Control antibodies had no effect (Figure 4A; Additional file 4). In a second approach, we used a soluble extracellular domain of p75^NTR ^to scavenge any p75^NTR ^ligands contained in the growth medium [25]. Again we observed a reduction in the level of repellent axon guidance (Figure 4B-G). These two independent sets of data suggest that striped outgrowth driven by ephrinA reverse signalling is dependent on local secretion of proneurotrophins, which induces an ephrinA-p75^NTR ^complex competent to 'read' repellent guidance signals. This complex is likely to contain also the sortilin receptor necessary for proBDNF-mediated p75^NTR ^functions [26]. Similarly, p75^NTR ^must be bound to its neurotrophin ligands to participate in Sema 3- and ephrinB2-mediated growth cone collapse of sympathetic neurons [27].

The ligand inducibility of this repulsion is of particular interest as it appears at first glance surprising that RGC axons can invade the tectum from the high end of the repellent EphA gradient (Additional file 3). In light of our findings, retinal axons conceivably become sensitive to the repellent gradient only after their ingrowth into the tectum, as possibly only then proBDNF is secreted from retinal axons leading to an induction of the ephrinA-p75^NTR ^complex. This switch from storage to secretion might be developmentally regulated and/or linked to the fact that proBDNF secretion is regulated by neural activity [13,15]. Thus, a change, for example, in the pattern of spontaneous neural activity might lead to proBDNF secretion only after RGC axons have invaded the tectum, that is, after they have surmounted the high end of the repellent EphA gradient.

### Antagonistic actions of proBDNF and BDNF on axonal branching

We have shown recently that a BDNF-promoted interaction between ephrinAs and TrkB results in an increase in retinal axon branching and that increasing the level of ephrinAs on retinal axons further increases the level of branching [6].

**Figure 5:**
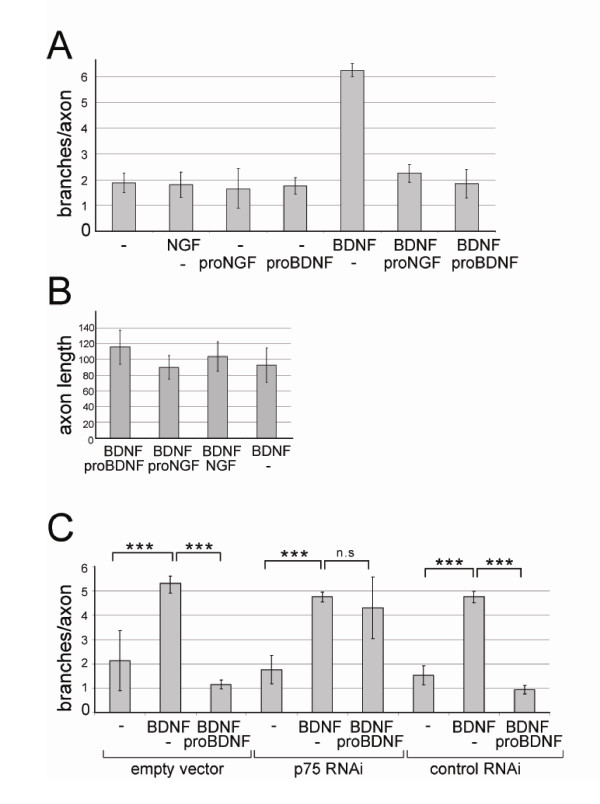
**Proneurotrophins abolish retinal axon branching via p75^NTR^**. The outgrowth/branching assay was performed as described [6]. Cells from E8 nasal retina were electroporated with eGFP and plated on a merosin/laminin substrate. **(A) **Cultures were treated at 1 day *in vitro *with 5 ng/ml BDNF, or 5 ng/ml proneurotrophins in the presence of 5 ng/ml BDNF as indicated, and fixed and analysed for branch number per axon after 3 days *in vitro*. The basal level of branching was not affected by treatment of retinal cultures with proneurotrophins alone. However, both proBDNF and proNGF led to a downregulation of the BDNF-induced branching to basal levels. **(B) **The length of outgrowth of retinal axons is not affected by treatment with neurotrophins and/or proneurotrophins. Axon length is given in arbitrary units. **(C) **To show that the proBDNF effect is mediated via p75^NTR^, retinal cultures were electroporated either with an RNAi vector resulting in the knockdown of p75^NTR^, with an RNAi vector not affecting p75^NTR ^protein levels or with empty vector. After plating, the cultures were treated with pro/neurotrophins as described in (A). p75^NTR ^knockdown obliterates the branch-suppressing effect of proBDNF. Three independent experiments were performed. The statistical analysis was done using Kruskal-Wallis test and Dunn's multiple comparison test *post hoc*. ***P < 0.001; n.s., not significant, the error bars represent S.E, M.

We have investigated now the role of the precursor form, proBDNF, on axon branching (Figure 5). We performed these experiments in the presence of 5 ng/ml BDNF to provide a substantial level of axon branching. Co-application of 5 ng/ml proBDNF then resulted in a down-regulation of axon branching, in that it effectively neutralised the branch-promoting effect of BDNF. Proneurotrophins alone had no effect on branching. This downregulation was mediated by p75^NTR ^as it was no longer observed after knock-down of this receptor (Figure 5C). A similar reduction in branching was observed when using a different proneurotrophin, proNGF, which does not interact with TrkB, neither in its pro- nor its processed form (Figure 5A). However, while mature NGF has no effect on RGC branching *in vivo *[28], we can not exclude that proNGF might be involved in suppressing RGC axon branching.

Thus, while BDNF application (via TrkB) promotes retinal branching, proBDNF application (via p75^NTR^) suppresses it. The map disturbance seen in (conditional) p75^NTR ^knockout mice with increased axonal branching anterior to the termination zone [5] is in good agreement with the data shown here, offering a molecular explanation for the observed p75^NTR ^phenotypes. Our data suggest that the local ratio between proBDNF and BDNF (and with that a differential activation of TrkB versus p75^NTR^) in the tectum/superior colliculus contributes to topographically specific branching. Thus, during map formation, proBDNF might be processed to BDNF only in those areas of the tectum where branching is topographically appropriate [21,29], leading to increased branching. Secretion and processing of proBNDF/BDNF from tectal cells might be involved here too.

## Conclusions

Our data on the antagonism of BDNF and proBDNF in retinotectal (nasal) axon guidance strengthens the general model of neurotrophin receptor function that proposes that antagonistic functions of Trks and p75^NTR ^control numerous biological processes [9,18]. Our data agree well, for example, with studies in the peripheral nervous system analysing the pruning of sympathetic axons after their projection to eye muscles. Here, correctly targeted ('winning') branches are maintained because of prevalent Trk signalling, while p75^NTR ^activation in 'losing' axons causes axonal degeneration by suppressing TrkA-mediated signalling [30,31].

## Materials and methods

### Experimental reagents

EphA7-Fc was from R&D Systems, Fc control from Calbiochem, BDNF from Promega, wild-type proBDNF and proNGF from Alomone Lab (Jerusalem, Israel). PanTrk203 antibody was a gift from Joan Comella, Lleida (Spain), and the antibody specific for the pro-domain of proBDNF (mAb287 [14]) was from Genecopoeia, Inc. (Rockville, MD, USA) Anti-FLAG and NGF was from Sigma and αHA from Abcam (Sigma: St. Louis, MO, USA; Abcam UK: Cambridge, U.K.). αHA and αFLAG agarose beads for immunoprecipitation of HA-tagged constructs were obtained from Sigma. The source of all other reagents is described in Marler *et al*. [6].

### Outgrowth/branching assays and stripe assays

Outgrowth/branching assays and stripe assays were performed as described in Marler *et al*. [6]. RGCs for both the stripe assay and the axon branching assay were grown in Neurobasal media with 2% B27 supplement, 1 mM glutamine, 5 μM forskolin and 1% penicillin/streptomycin; thus, in synthetic media containing, *per se*, no neurotrophins. For the axon branching assay the media were supplemented with proneurotrophins or neurotrophins as described in the respective figure legends. RGCs were identified by staining for markers expressed on retinal axons, such as TrkB, Brn3A and Thy-I. Routinely, those cells with the longest axons were positive for these markers. Scoring was performed after 3 days for the branching assay using proneurotrophin/neurotrophin treatment (Figure 5). For the standard stripe assay, E6 retinae were used and scoring was performed after 2 days in culture. Here the cells were plated on a substrate of alternating lanes of EphA7-Fc and Fc, or Fc and Fc as a control. Protocols are described in Marler *et al*. [6] and Rashid *et al*. [4]. To investigate axon guidance in the stripe assay, concentrations of 30 μg/ml each were used. Axons were scored as '0' if the majority of axons showed no preference for either stripe. They were scored as '1' if they showed some avoidance of the EphA7-Fc* stripe (or Fc*), and as '2' for a strong preference for growing on Fc stripes, that is, almost completely or fully avoided the EphA7-Fc* (or Fc*) stripes. The evaluation of all branching and stripe assays was done blind to the constructs transfected and ligands added. Stripe assay data shown are the result of three independently performed experiments.

For the proBDNF stripe assay experiments, single cells from E6 chick retina were electroporated with an eGFP expression plasmid and plated on alternating lanes of EphA7-Fc and Fc, or Fc and Fc. Experiments were performed in the presence of a proBDNF antibody (1:200) [14] or a control antibody (mouse monoclonal antibody for placental alkaline phosphatase; 1:200). The quantification of axon growth preferences shows an abolishment of repellent guidance in the presence of the proBDNF antibody. The number of axons analysed for each experimental condition was > 40.

For the axonal branching assays single cells were prepared from the nasal third of E8 chick retinae and electroporated with an eGFP expression construct to facilitate later analysis of neurite outgrowth. Cultures were plated on laminin (10 μg/ml) and merosin (2 μg/ml) coated dishes and cultured for 4 days.

### Co-immunoprecipitation

Co-immunoprecipitation experiments were performed as described in Marler *et al*. [6]. In brief, after transfection, cells were lysed in 25 mM Tris, pH 7.4, 150 mM NaCl, 1% (v/v) Triton X100, 1 mM EDTA pH 8.0, 10% (v/v) glycerol, 1 mM phenylmethyl-sulfonyl fluoride (PMSF), 1 mM sodium fluoride (NaF), 1 mM sodium vanadate, 1 mM sodium pyrophosphate, 1 mM glycerol pyrophosphate and Roche Complete Protease Inhibitor. (Roche UK: Hertfordshire, U.K.). After passing through a 26G needle, cell lysates were cleared by centrifugation for 20 minutes at 13,000 g. Approximately 1 mg of total protein from each sample was incubated with 20 μl of respective affinity gel overnight at 4°C on an orbital shaker. Immunocomplexes were washed four times with lysis buffer, suspended in 20 μl of Laemmli buffer, boiled and loaded onto a 10% SDS-polyacrylamide gel, and transferred onto nitrocellulose. Blots were immunodetected with suitable antibodies and developed using Western Lightning' Chemoluminescence Reagent from Perkin Elmer according to the manufacturer's instructions and it was exposed to an X-ray film as long as required (Perkin Elmer: Waltham, MA, USA).

### RNAi knockdown

RNAi knockdown experiments were performed as described in Marler *et al*. [6]. Target sequences for p75^NTR ^RNAi(2) were ACCTCCGATGCTGAGTGCAGA and for p75^NTR ^RNAi(3) were CTACGGCTACTTCCAGGATG.

### Cloning and expression of soluble p75^NTR, sol, FLAG ^DNA

The extracellular domain of p75^NTR ^was obtained by PCR using the forward primer CC TGC CTG GAC AGT GTG, and reverse primer TCA GGT GCC ACG GCT CAC, covering the amino acids PCLDS.....VSRGT, using the chick p75^NTR ^cDNA as a template. The resultant PCR fragment was cloned into a CMV promoter-containing expression vector with 5' sequences encoding a signal peptide and a triplicate FLAG tag. Clones were sequence-verified. For stripe assay experiments involving p75^NTR, sol, FLAG^, two 6 cm^2 ^plates with 5 × 10^5 ^CHO cells each were transfected per construct. Two days later the media were harvested and 500 μl of transfected or mock transfected media was added to each of the stripe assay cover slips to give a 1:1 ratio. A further 1 ml of the media was reserved and analysed for the presence of p75^NTR, sol, FLAG^.

## Abbreviations

BDNF: brain derived neurotrophic factor; CHO: Chinese hamster ovary; E: embryonic day; eGFP: enhanced green fluorescent protein; NGF: nerve growth factor; NTR: neurotrophin receptor; RGC: retinal ganglion cells; RNAi: RNA interference; Trk: tropomyosin-related kinase.

## Competing interests

The authors declare that they have no competing interests.

## Authors' contributions

KM planned and performed experiments, to which SP, EB and CW contributed. CW characterised the p75 RNAi vectors. UD planned the experiments, supervised the project and wrote the paper.

## Supplementary Material

Additional file 1 **Supplemental Figure **1. The ephrinA5-p75^NTR ^interaction is promoted in a proneurotrophin-dependent manner. **(A) **CHO cells were transfected with plasmids encoding ephrinA5^FLAG ^and TrkA^HA ^or p75^NTR;HA^. One day later cells were lysed and subjected to immunoprecipitation using a αFLAG antibody and analysed in western blots as indicated. At low expression levels, ephrinA5 co-immunoprecipitates with TrkA but not p75^NTR^. IB, immunoblot; IP, immunoprecipitation. **(B) **CHO cells were transfected with p75^NTR, FLAG ^and ephrinA5^HA. ^A day later cells were serum starved and treated for 30 minutes with pro/neurotrophins as indicated. Subsequently, cells were lysed and immunoprecipitated using a αHA antibody. Western blots showed that co-immunoprecipitation of p75^NTR ^with ephrinA5 is increased in the presence of ligand. Quantification is shown in Additional file 2. Click here for file

Additional file 2 **Supplemental Figure **2. Quantification of ligand-induced co-immunoprecipitation of p75^NTR ^with ephrinA5^HA^. Quantification of co-immunoprecipitation experiments as exemplified in Figure 1 and Additional file 1. For experimental details see legends for Figure 1 and Additional file 1. Concentrations used for the co-immunoprecipitations are given. The numbers of independently performed experiments were: for NGF, n = 4; for proNGF n = 4; and for proBDNF n = 3. For quantification, the intensity of bands corresponding to immunoprecipitated p75^NTR ^was normalised using the intensity of bands corresponding to ephrinA5^HA^. Then ratios were determined between values obtained for presence versus absence of ligand. In the absence of ligand (control) the value is 1. The standard error of the mean is shown. Click here for file

Additional file 3 **Supplemental Figure **3. The projection patterns of RGC axons from the retina to the tectum, the differential expression patterns of EphAs and ephrinAs in retina and tectum, as well as the uniform expression the neurotrophin receptors TrkB and p75^NTR ^and their ligands in the retina. Click here for file

Additional file 4 **Supplemental Figure **4. Abolishment of striped outgrowth of RGC axons on an EphA7-Fc/Fc matrix in the presence of a proBDNF antibody. **(A) **In the presence of a control antibody, a RGC axon (green) avoids a lane containing EphA7-Fc (in red). **(B) **In the presence of the proBDNF antibody, a RGC axon freely crosses EphA7-Fc (red) and Fc (unlabelled) lanes. Details of the experimental conditions are described in Figure 4A. Scale bar = 25 μm. Click here for file
