# Supplementary figures and images for: Pro-neurotrophins secreted from retinal ganglion cell axons are necessary for ephrinA-p75NTR-mediated axon guidance
Source: Neural Dev. 2010 Nov 2;5:30. doi: 10.1186/1749-8104-5-30 (PMC2987844; doi:10.1186/1749-8104-5-30)

A

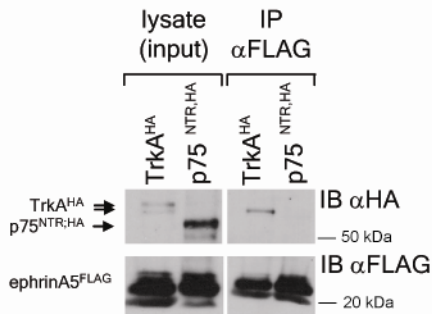

B

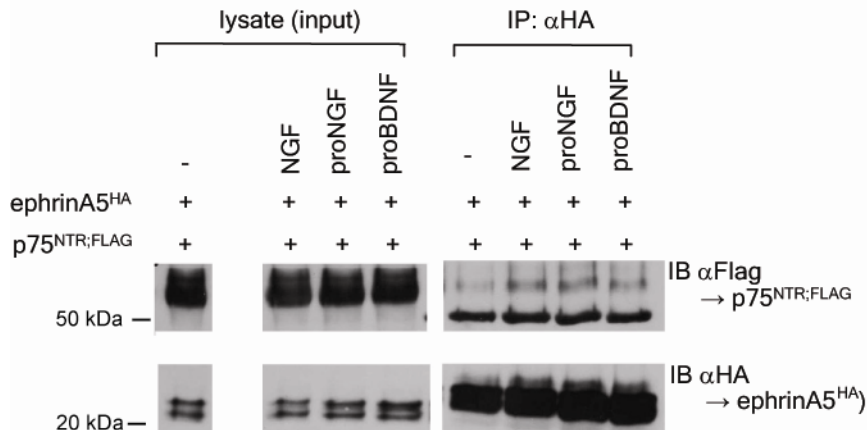

Supplement: Additional file 1 — Supplemental Figure 1. The ephrinA5-p75NTR interaction is promoted in a proneurotrophin-dependent manner. (A) CHO cells were transfected with plasmids encoding ephrinA5FLAG and TrkAHA or p75NTR;HA. One day later cells were lysed and subjected to immunoprecipitation using a αFLAG antibody and analysed in western blots as indicated. At low expression levels, ephrinA5 co-immunoprecipitates with TrkA but not p75NTR. IB, immunoblot; IP, immunoprecipitation. (B) CHO cells were transfected with p75NTR, FLAG and ephrinA5HA. A day later cells were serum starved and treated for 30 minutes with pro/neurotrophins as indicated. Subsequently, cells were lysed and immunoprecipitated using a αHA antibody. Western blots showed that co-immunoprecipitation of p75NTR with ephrinA5 is increased in the presence of ligand. Quantification is shown in Additional file 2. [file 1749-8104-5-30-S1.PDF]

arbitrary units

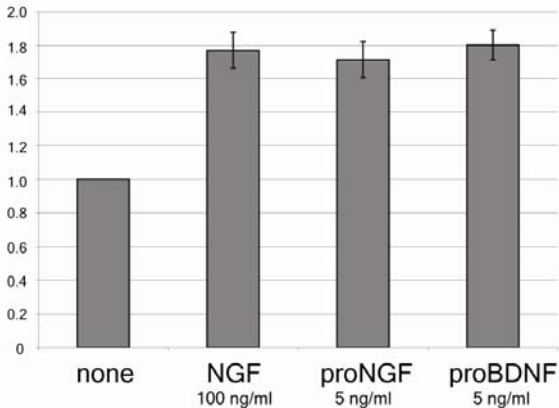

Supplement: Additional file 2 — Supplemental Figure 2. Quantification of ligand-induced co-immunoprecipitation of p75NTR with ephrinA5HA. Quantification of co-immunoprecipitation experiments as exemplified in Figure 1 and Additional file 1. For experimental details see legends for Figure 1 and Additional file 1. Concentrations used for the co-immunoprecipitations are given. The numbers of independently performed experiments were: for NGF, n = 4; for proNGF n = 4; and for proBDNF n = 3. For quantification, the intensity of bands corresponding to immunoprecipitated p75NTR was normalised using the intensity of bands corresponding to ephrinA5HA. Then ratios were determined between values obtained for presence versus absence of ligand. In the absence of ligand (control) the value is 1. The standard error of the mean is shown. [file 1749-8104-5-30-S2.PDF]

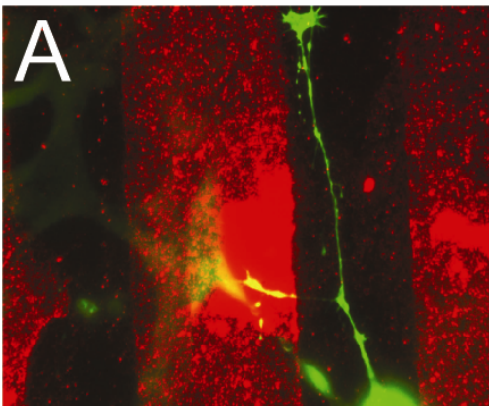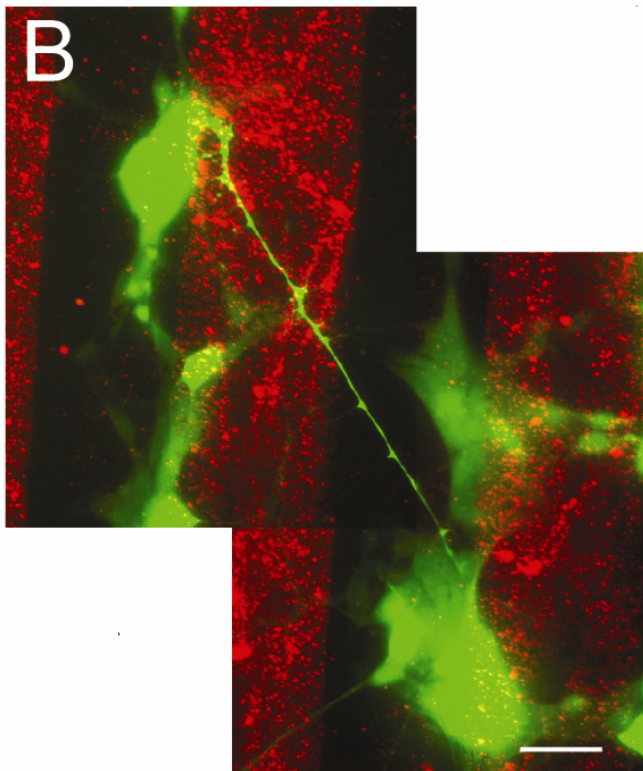

Supplement: Additional file 4 — Supplemental Figure 4. Abolishment of striped outgrowth of RGC axons on an EphA7-Fc/Fc matrix in the presence of a proBDNF antibody. (A) In the presence of a control antibody, a RGC axon (green) avoids a lane containing EphA7-Fc (in red). (B) In the presence of the proBDNF antibody, a RGC axon freely crosses EphA7-Fc (red) and Fc (unlabelled) lanes. Details of the experimental conditions are described in Figure 4A. Scale bar = 25 μm. [file 1749-8104-5-30-S4.PDF]
