# Supplementary material for: Pro-neurotrophins secreted from retinal ganglion cell axons are necessary for ephrinA-p75NTR-mediated axon guidance
Source: Neural Dev. 2010 Nov 2;5:30. doi: 10.1186/1749-8104-5-30 (PMC2987844; doi:10.1186/1749-8104-5-30)
Supplement: Additional file 3 — Supplemental Figure 3. The projection patterns of RGC axons from the retina to the tectum, the differential expression patterns of EphAs and ephrinAs in retina and tectum, as well as the uniform expression the neurotrophin receptors TrkB and p75NTR and their ligands in the retina. [file 1749-8104-5-30-S3.PDF]

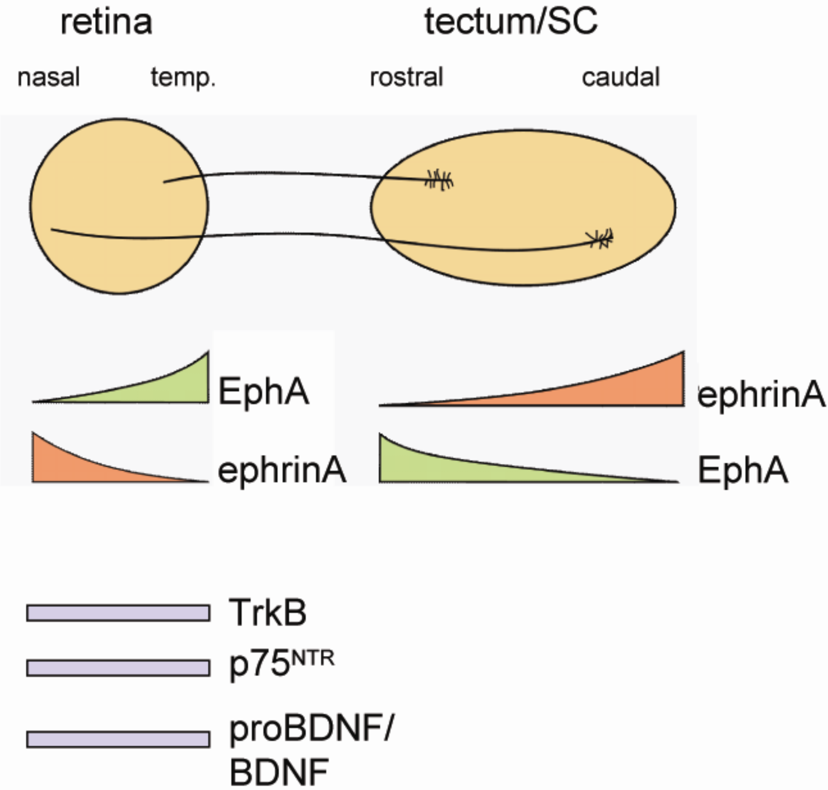
retina
tectum/SC
nasal
temp.
rostral
caudal
EphA
ephrinA
ephrinA
EphA
TrkB
p75NTR
proBDNF/
BDNF
